# Twinning as a risk factor for neonatal acute intestinal diseases: a case-control study

**DOI:** 10.3389/fped.2023.1308538

**Published:** 2023-12-14

**Authors:** C. Peila, E. Spada, L. Riboldi, M. Capitanio, F. Pellegrino, A. Coscia

**Affiliations:** ^1^ Neonatal Intensive Care Unit, Department of Public Health and Pediatrics, University of Turin, Turin, Italy; ^2^ Laboratorio Della Conoscenza Carlo Corchia—APS, Florence, Italy

**Keywords:** acute intestinal diseases, necrotizing enterocolitis, spontaneous intestinal perforation, twin, twinning, preterm newborn, newborn

## Abstract

**Introduction:**

Acute intestinal diseases (AID), including necrotizing enterocolitis and spontaneous intestinal perforation, are a group of conditions that typically present in preterm infants, and are associated with an elevated mortality and morbidity rate. The risk factors for these diseases remain largely unknown. The aim of the study is to identify the correlation between twinning and the development of AID.

**Methods:**

A single-center retrospective case–control study was conducted. We recruited all infants with a diagnosis of AID, confirmed by anatomopathology, recovered in NICU between 2010 and 2020. Considering the rarity of the outcome, 4 matched controls for each subject were randomly chosen from the overall population of newborns. Odds Ratio (OR) and 95% Confidence Interval (CI) were calculated using a conditional logistic regression model and a multivariate model by the creation of a Directed Acyclic Graph (www.dagitty.net).

**Results:**

The study population resulted in 65 cases and 260 controls. The two groups present similar median gestational age and mean birthweight in grams. The cases have a higher frequency of neonatal pathology (defined as at least one of patent ductus arteriosus, early or late sepsis, severe respiratory distress) (84.6% vs. 51.9%), medically assisted procreation (33.8% vs. 18.8%) and periventricular leukomalacia (10.8% vs. 2.7%), and a lower frequency of steroids prophylaxis (67.7% vs. 86.9%). About 50% of cases needed surgery. The OR for the direct effect were difference from one using logistic regression booth without and with repeated measures statements: from 1.14 to 4.21 (*p* = .019) and from 1.16 to 4.29 (*p* = .016), respectively.

**Conclusions:**

Our study suggests that twinning may be a risk factor for the development of AID. Due to the small number of cases observed, further studies on larger populations are needed.

## 1. Introduction

Neonatal acute intestinal diseases (AID) are surgical intestinal disorders without mechanical obstruction typical of preterm newborns, including necrotizing enterocolitis (NEC) and spontaneous intestinal perforation (SIP). Despite recent advancements in neonatal care, these surgical intestinal disorders are still associated with a high mortality rate and a high prevalence of long-term morbidity in affected preterm infants.

NEC is the most frequent disease of the gastrointestinal tract of preterm infants and represents the most common cause of mortality and morbidity in the Neonatal Intensive Care Unit (NICU) (1, 2). Multiple population-based studies have reported the incidence of NEC to vary from 2 to 13% in NICU population, although there is substantial variability in incidence reported from different parts of the world (3, 4). Prognosis of NEC is related to gestational age (GA), with an estimated overall mortality from confirmed NEC of 25%, rising to 50% in Extremely Low Birth Weight (ELBW) infants (5). Pathogenesis of NEC is still partially unknown, but certainly multifactorial. The clinical presentation can be insidious or fulminant. Treatments involve supportive clinical management and consist of stopping enteral feedings and providing parenteral nutrition, intestinal decompression by nasogastric suctioning, and empiric administration of broad-spectrum antibiotics. In severe cases, surgical management often consists of peritoneal drain placement or exploratory laparotomy with possible bowel resection and percutaneous enterostomy placement (1).

SIP, characterized by the presence of focal intestinal perforation with no or minimal adjacent bowel inflammation, has confirmed to be a separate disease entity from NEC, because necrosis, inflammation of the intestinal mucosa or alterations in blood flow are generally not observed (6). Mortality rate is lower in infants with SIP in comparison with those with NEC, however these patients are particularly fragile and susceptible to short- and long-term complications (7, 8). SIP is a surgical disease, and the treatment is based on two main options currently used: exploratory laparotomy with bowel resection, or peritoneal drainage, which can be used either as a stabilizing procedure or a definitive treatment. Another minimally invasive and less used option is peritoneal needle aspiration (9).

It is therefore important to identify as many AID risk factors as possible in order to prevent them or, where the risk is unavoidable, perform early intervention. Regarding SIP risk factor, few studies have been conducted on this topic and there are not many certainties. On the other hand, for NEC risk factors, several associations were investigated and classified in antenatal, perinatal and postnatal. Established risks include prematurity and low birth weight (5).

Other certain risk factors reported currently in Literature are: prolonged rupture of membranes and maternal chorioamnionitis; compromised fetal blood flow before or at the time of delivery that may result in fetal ischemia; Intrauterine growth restriction (IUGR) especially if associated with abnormal Doppler studies; many typical complications associated with prematurity and some medication routinely administered to NICU's patients (sepsis, anemia, Patent ductus arteriosus); bacterial colonization of the gut and formula-feeding (5, 10–23).

In addition to the already known risk factors, clinical observations, supported by a plausible biological explanation, suggest that twinning may be associated with the risk of AID. In fact, many studies outlined that twin pregnancies present alterations not only in placental but also in fetal microcirculation with a greater frequency than that observed in single pregnancies. Those alterations of the microcirculatory perfusion of gastrointestinal tract, before or at the time of delivery, may lead to intestinal diseases development (24, 25).

The aim of this study is to identify a possible association between twinning and the development of NEC and/or SIP.

## 2. Methods

The study population was extracted from the St. Anne's Hospital dataset and selected from infants born between 2010 and 2020 admitted to the neonatal intensive care unit. All cases -defined as infants who developed NEC or SIP from birth to discharge- were collected. For each case, 4 controls -defined as infants who did not develop NEC or SIP from birth to discharge- were randomly extracted, with a final case:controls ratio of 1:4.

In the description of the sample, the categorical variables were presented as frequencies (percent), while the continuous variables were presented as mean (standard deviation) or median (interquartile range) according to their distribution.

**Figure 1:**
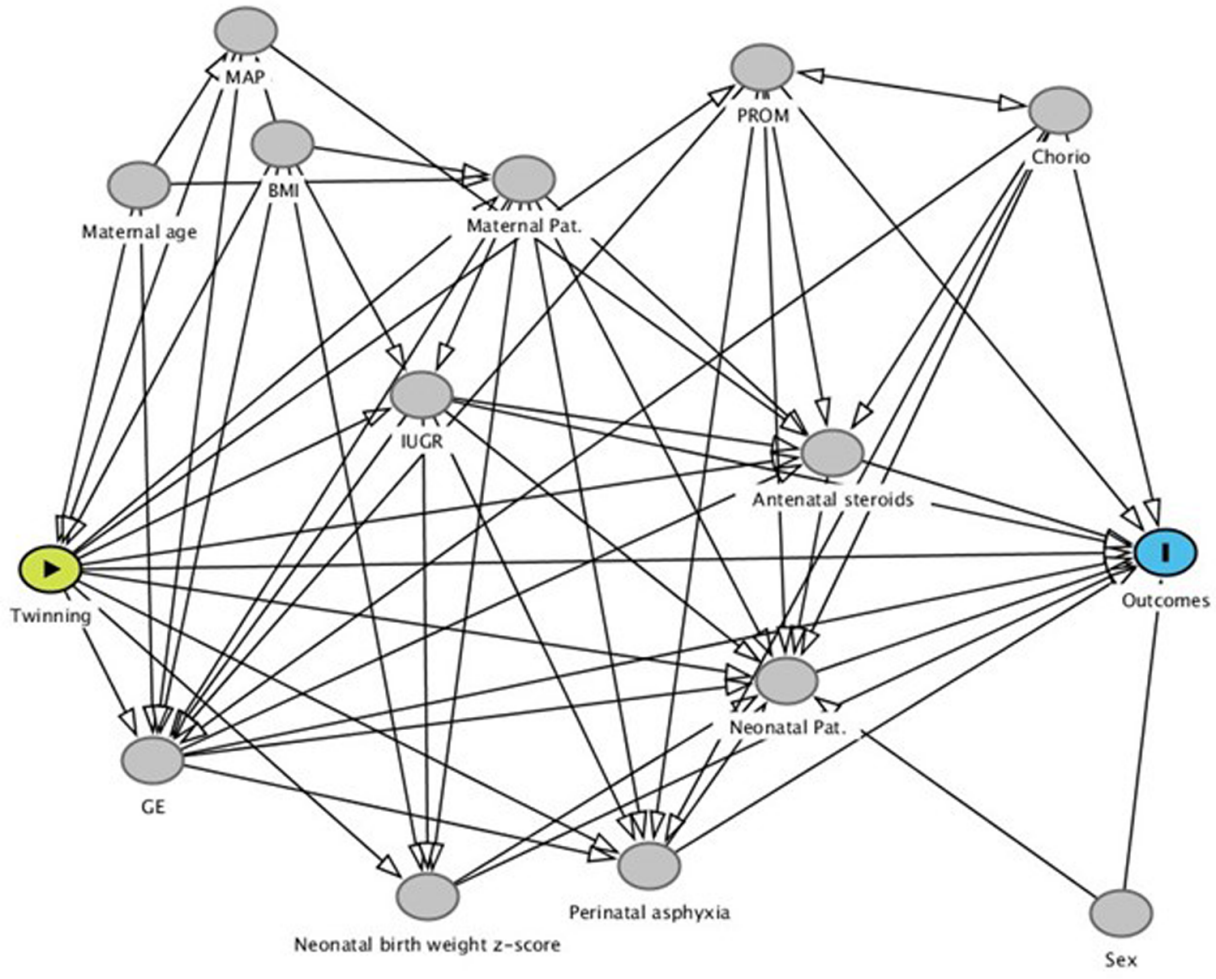
Directed acyclic graph. This graph helps in visualizing the relationships between the variables and in allows to identify the covariate for the estimate of direct and total effect.

The effect of twinning was analyzed using a logistic regression. The use of DAG allowed to identify the covariate for the estimate of direct and total effect; where the direct effect is the effect of twinning vs. singleton all the other covariate been equal, i.e., the effect added by twinning alone, and total effect is the effect of twinning vs. singleton, i.e., direct plus mediate by other variables (as gestational age) effect (Figure 1). The adjustment variables for direct effect were sex, gestational age (weeks), birth weight (z-score using INeS charts as reference) (26), intra uterine growth restriction, chorioamnionitis, premature rupture of membranes, neonatal pathology (defined as at least one of patent ductus arteriosus, early or late sepsis, severe respiratory distress), perinatal asphyxia, steroid prophylaxis (at least one cycles). The adjustment variables for total effect were maternal obesity (BMI ≥ 35 kg/m^2^), medically assisted procreation, maternal age.

A further analysis was performed including the repeated statement in logistic regression to model the covariance between the twin siblings, considering the couples of twins' outcomes repeated measures.

Only records with missing values of one or more variables included in the models were excluded.

The reference group was control group.

## 3. Results

From 2010 to 2020, 72 neonates admitted at St. Anne's Hospital NICU were affected by NEC or SIP. From these 7 (8.9%) neonates were excluded due to missing maternal values (BMI and age). Their gestational age ranged from 24 to 34 weeks, 1 mother had medically assisted procreation, 3 (43%) were boys. The study population resulted in 325 newborns (65 cases and 260 controls) and the description of the two groups is reported in Table 1.

**Table 1:** Description of neonatal and mother characteristics of cases and controls.

	Cases	Controls
	(*n* = 65)	(*n* = 260)
Neonatal characteristics		
Boys, *n* (%)	33 (50.8)	119 (45.8)
Birth weight (g), mean (SD)	1,108 (545)	1,194 (637)
Birth weight (z-score^a^), mean (SD)	−0.14 (0.98)	−0.06 (0.99)
SGA^b^, *n* (%)	8 (12.3)	40 (15.4)
LGA^b^, *n* (%)	2 (3.1)	15 (5.8)
Gestational Age (weeks), median (IQR)	28 (25–30)	28 (26–30)
Extremely preterm^c^, *n* (%)	31 (47.7)	115 (44.2)
Very Preterm^c^, *n* (%)	23 (35.4)	91 (35.0)
Moderate to late preterm^c^, *n* (%)	9 (13.4)	43 (16.5)
Vaginal delivery, *n* (%)	21 (32.3)	101 (38.8)
Twins, *n* (%)	30 (46.2)	91 (35.0)
Respiratory Distress Syndrome, *n* (%)	54 (83.1)	225 (86.5)
High frequency oscillatory ventilation	11 (16.9)	22 (8.5)
Patent ductus arteriosus	44 (67.7)	120 (46.2)
Early sepsis	2 (3.1)	11 (4.2)
Late sepsis	28 (43.1)	23 (8.9)
Inhaled nitric oxide	7 (10.8)	12 (4.6)
Neonatal pathology^d^, *n* (%)	55 (84.6)	135 (51.9)
Surfactant, *n* (%)	40 (61.5)	197 (75.8)
Retinopathy of prematurity	12 (18.5)	62 (23.9)
Intraventricular Hemorrhage	13 (20.0)	42 (16.2)
Periventricular Leukomalacia, *n* (%)	7 (10.8)	7 (2.7)
Perinatal Asphyxia, *n* (%)	20 (30.8)	34 (13.1)
Perinatal distress, *n* (%)	20 (30.8)	34 (13.1)
Delayed Feeding start^e^, *n* (%)	11 (16.9)	56 (21.5)
Feeding Intolerance^f^, *n* (%)	39 (60)	45 (17.3)
Maternal characteristics		
Age (years), median (IQR)	36 (31–38)	35 (30–38)
BMI, mean (SD)	23.7 (3.6)	24.3 (5.5)
Obesity (BMI ≥ 35 kg/m^2^), *n* (%)	1 (1.5)	14 (5.4)
Gestational diabetes, *n* (%)	7 (10.8)	36 (13.8)
Hypertension, *n* (%)	16 (24.6)	67 (25.8)
Medically assisted procreation, *n* (%)	22 (33.8)	49 (18.8)
Intra Uterine Growth Restriction, *n* (%)	12 (18.5)	60 (23.1)
Premature rupture of membranes, *n* (%)	18 (27.7)	96 (36.9)
Chorioamnionitis, *n* (%)	15 (23.1)	60 (23.1)
Steroid prophylaxis, *n* (%)	44 (67.7)	226 (86.9)

^a^ According with INeS reference. The reference for twins is firstborn charts.

^b^ SGA: birth weight z-score < −1.28; LGA: birth weight z-score > +1.28.

^c^ extremely preterm: GA < 28 weeks; preterm: GA between 28 and 31 weeks; moderate to late preterm: GA between 32 and 36 weeks.

^d^ neonatal pathology: at least one of patent ductus arteriosus, early or late sepsis, severe respiratory distress.

^e^ Delayed feeding start: delayed start of nutrition over 48 h of life.

^f^ Feeding intolerance: at least one episode requiring >24 h suspension of nutrition.

The median gestational age is similar. In cases the frequency of boys is slightly higher while the frequency of twins is higher of 10% (46.2% cases vs. 35.0% controls). The two groups present similar mean birthweight in grams, but a mean z-score lower in case than in control group. The cases have a higher frequency of neonatal pathology (defined as at least one of patent ductus arteriosus, early or late sepsis, severe respiratory distress) (84.6% cases vs. 51.9% controls), medically assisted procreation (33.8% cases vs. 18.8% controls) and periventricular leukomalacia (10.8% cases vs. 2.7% controls), and a lower frequency of intrauterine growth restriction (18.5% cases vs. 23.1%controls) and steroids prophylaxis (67.7% cases vs. 86.9% controls). While early sepsis is comparable between the two groups (3.1% cases vs. 4.2% controls), the frequency of late sepsis is more than 5 times in cases than in controls (43.1% cases vs. 8.9% controls). About 30% of cases presented perinatal distress vs. 13% of controls.

Table 2 reports the frequencies of NEC, SIP and need for surgery for these conditions in cases. About 50% of cases needed surgery.

**Table 2:** Number (frequency) of gastrointestinal diseases between cases.

Cases (*n* = 65)	
Medical Necrotizing Enterocolitis	16 (24.6)
Surgical Necrotizing Enterocolitis	32 (49.2)
Spontaneous Intestinal Perforation	17 (26.2)

**Figure 2:**
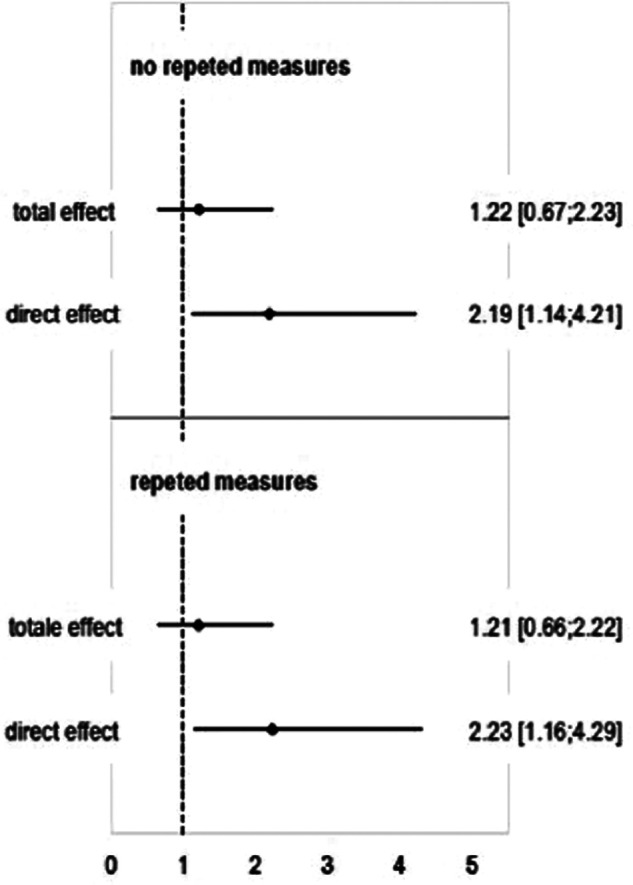
Direct and total effect ORs [95% CI] estimated using logistic regression and logistic regression with repeated measures (i.e. the 21 pairs of twins were considered as repeat measures in order to account for covariance between twin siblings).

The Odds Ratio estimates are reported in Figure 2. While the total effect does not show significant difference, the ORs for the direct effect were difference from one using logistic regression booth without and with repeated measures statement: from 1.14 to 4.21 (*p* = .019) and from 1.16 to 4.29 (*p* = .016), respectively.

## 4. Discussion

In last decades, the improvement of neonatal techniques has allowed a greater survival of premature infants, with increasingly satisfying long-term outcomes. Despite this recent advancement, incidence of AID has remained relatively stable in infant populations with a high risk of mortality and significant short- and long-term morbidity (27, 28).

The management of AID has remained almost stable over the years, so prevention and identification of the risk factors are mandatory.

In Literature, many risk factors have been analyzed and confirmed by recent studies, while others are still controversial or not fully considered (5, 10–23). Few studies considered the possibility that twinning was a yet unknown risk factor, despite its clinical and biological plausibility. Literature data are mainly focused on the NEC and twinning has been used as a paradigm in the understanding of etiopathogenesis (29–31). Conversely, in studies focused on specific types of twin pregnancy, NEC is considered as one of the neonatal morbidities under analysis and in these cases an association was observed (32, 33).

Regarding the characteristics of our two populations of cases and controls, the main data observed were in line with the literature. Among population of cases, we observed a higher frequency of some neonatal risk factors associated with AID:

- perinatal asphyxia: poor neonatal transition
- PDA: an association between the presence of PDA and the development of intestinal surgical pathologies seems to emerge from numerous studies in recent years (15, 34).
- Sepsis: the same can be said of the association between septic pathology of the newborn and the risk of developing NEC (10, 20)
- Severe RDS (20, 33, 35)
- Male sex (33)

In recent years, a possible protective role of prenatal administration of corticosteroids towards the development of NEC has emerged, and also in our population we observed a lower frequency of steroids prophylaxis in cases compared to controls.

It is also interesting to note the higher number of assisted fertilization pregnancies among cases. This observation can be explained considering that the pregnancies obtained with medically assisted procreation are often at risk for preterm labor and pre or postnatal complications, and they generate twin pregnancies more frequently than spontaneous.

The core of our study is the relationship between twinning and the development of AID. The construction of a DAG allowed us to analyze this association without considering the confounding factors that could modify the association between the risk factor and the outcome.

This data is extremely interesting because it seems to indicate that twinning is not only a risk factor of premature birth, *per se* known as a major risk factor for the onset of AID in the newborn, but that it constitutes in itself a direct risk factor of AID.

The ORs for direct effect resulted higher respect to ORs for total effect. This result can be explained from the different distribution of variables included in multivariable analysis: lower z-score and steroids prophylaxis, intrauterine growth restriction, premature rupture of membranes, and higher perinatal distress.

The case-control study has the advantage of allowing us to have information on the association between two variables in a short time and is useful for exploratory purposes, especially when the risk is rare. Like all studies with retrospective data collection, whether prospective or case-control, the major disadvantage is related to the possible bias due to collected variables not specifically for the purpose. However, it must be considered that this affordability is the same for clinical purposes only. The main limitation of the study lies in the sample size that is about 30%. Despite this, the direct effect results significant. Future studies, possibly multicentric, are needed to expand the sample size and confirm our results. In future studies, it would also be interesting to collect more data related to both twins, regardless of whether they are both cases and/or controls to have a complete picture of the twin population considered. Finally, it would also be important to collect detailed data related to nutrition (i.e., type of nutrition, method of nutrition administration, the rhythm of increasing the amount of meal, day of life for beginning the nutrition) because in a multicenter study the nutritional protocols may differ substantially between centers, and then these variables will also be included in the multivariate analysis.

## 5. Conclusions

AID still remains one of the leading causes of death in preterm infants, with still largely unclear characteristics. The ever more in-depth knowledge of the risk factors that favor the onset of these diseases has made it possible to prevent and early recognize them. Twinning was not yet included in the group of the recognized risk factor, and few studies considered it before. This study allowed to identify an association between twinning and the development of NEC and SIP, providing a deeper knowledge of these diseases and a possibility of prevention for the future. Given the still small number of the sample analyzed, it is desirable that studies conducted on larger populations allow to confirm the observed data and to deepen it in the future.

## Data Availability

The raw data supporting the conclusions of this article will be made available by the authors, without undue reservation.
